# Xanthine Oxidase Inhibitory Activity, Chemical Composition, Antioxidant Properties and GC-MS Analysis of Keladi Candik (*Alocasia longiloba* Miq)

**DOI:** 10.3390/molecules25112658

**Published:** 2020-06-08

**Authors:** Ferid Abdulhafiz, Arifullah Mohammed, Fatimah Kayat, Matcha Bhaskar, Zulhazman Hamzah, Sanjay Kumar Podapati, Lebaka Veeranjaneya Reddy

**Affiliations:** 1 Faculty of Agro-Based Industry, University Malaysia Kelantan, Jeli Campus, Jeli 17600, Kelantan, Malaysia; feridabdul24@gmail.com (F.A.); fatimah@umk.edu.my (F.K.); sanjay.biochem@gmail.com (S.K.P.); 2 Institute of Food Security and Sustainable Agriculture (IFSSA), University Malaysia Kelantan, Jeli 17600, Kelantan, Malaysia; 3 Division of Animal Biotechnology, Department of Zoology, Sri Venkateswara University, Tirupati, Andhra Pradesh 517502, India; matchabhaskar@gmail.com; 4 Faculty of Earth Science, University Malaysia Kelantan, Jeli 17600, Kelantan, Malaysia; zulhazman@umk.edu.my; 5 Department of Microbiology, Yogi Vemana University, Kadapa, Andhra Pradesh 516003, India; lvereddy@yahoo.com

**Keywords:** gout, xanthine oxidase inhibitors, hyperuricemia, keladi candik, ethanol extract

## Abstract

*Alocasia longiloba*, locally known as ‘Keladi Candik’, has been used traditionally to treat wounds, furuncle and joint inflammations. *A. longiloba* can be a new source of herbal medicine against hyperuricemia by inhibiting the activity of xanthine oxidase enzyme, the enzyme which is responsible for the development of hyperuricemia in human. Existing xanthine oxidase inhibitors (XOI drugs) show several side effects on gout patients. Therefore, an alternative herbal medicine from plants, with high therapeutic property and free of side effects, are greatly needed. This study was conducted to evaluate XO inhibitory activity, chemical composition, antioxidant activity and GC-MS profile of *A. longiloba*. Our results showed that ethanolic petiole extract exhibited the highest XO inhibitory activity (70.40 ± 0.05%) with IC_50_ value of 42.71 μg/mL, followed by ethanolic fruit extracts (61.44 ± 1.24%) with the IC_50_ value of 51.32 μg/mL. In a parallel study, the phytochemical analysis showed the presence of alkaloid, flavonoid, terpenoids, glycoside and saponin in petiole and fruit extracts, as well as higher total phenolic and flavonoid contents and strong scavenging activity on DPPH and ABTS antioxidant assay. The GC-MS analysis of fruit and petiole extracts revealed the presence of various compounds belonging to different chemical nature, among them are limonen-6-ol, α-DGlucopyranoside, paromomycin, aziridine, phenol, Heptatriacotanol, Phen-1,2,3-dimethyl and Betulin found in ethanolic fruit extract, and Phen-1,4-diol,2,3-dimethyl-, 1-Ethynyl-3,trans(1,1-dimethylethyl), Phenol,2,6-dimethoxy-4-(2-propenyl)- and 7-Methyl-Z-tetradecen-1-olacetate found in ethanolic petiole extract. Some compounds were documented as potent anti-inflammatory and arthritis related diseases by other researchers. In this study, the efficiency of solvents to extract bioactives was found to be ethanol > water, methanol > hexane > chloroform. Together, our results suggest the prospective utilization of fruit and petiole of *A. longiloba* to inhibit the activity of XO enzyme.

## 1. Introduction

Medicinal plants (herbal medicines) have been used worldwide as an alternative and/or a complementary medicines. The use of herbal medicine has vastly increased due to their efficacy, affordability, availability and safety claims. The world health organization reported that 80–90% of the population in the developing countries relies on plant based medicines as a primary health care modality [1,2]. Medicinal plants are rich in a variety of important phytochemicals (active compounds), many are secondary metabolites, such as alkaloids, flavonoids, terpenoids, saponins and many others. These compounds possess antioxidant properties that are important for pharmaceuticals and drug development, as well as direct use as therapeutic agents. Antioxidants play a major role in preventing oxidation of molecules inside a cell and protecting healthy cells from damage caused by free radicals, unstable and highly reactive molecules that the human body produces as a byproduct of metabolism and other pressures. Therefore, protection against oxidative damage/cell death is the pivotal mechanism for preventing the occurrence of most diseases [3,4,5].

Gout is a form of inflammatory arthritis caused by high concentration of uric acid in the bloodstream. Supersaturation of uric acid (hyperuricemia) may lead to a uric acid crystals accumulation and deposition in the joint and tissue to which the immune system responds. Augmentation of urate in the body occurs as a result of excessive purine intake or metabolism defects and/or insufficient uric acid excretion. Xanthine oxidase is a type of enzyme responsible for the formation of uric acid in human body. This enzyme catalyzes the oxidation of hypoxanthine to xanthine and subsequently to uric acid [6]. Uric acid is primarily produced in the liver and then excreted by the kidneys into the urine [7]. Recent studies by several researchers, reported that accumulation of urate crystals can cause swelling, heat and joint pain, which are the main manifestation of gout disease [7,8,9].

Current medications used to treat gout disease mainly by using xanthine oxidase inhibitor (XOI) drugs such as allopurinol, steroids and non-steroidal anti-inflammatory, which can reduce the formation of uric acid or increase the kidney′s ability to eliminate uric acid from the body [10]. The mechanism of action of XOI is either by acting at the purine binding site [11] or at the flavin adenine dinucleotide co-factor site [12]. XOI drugs can also block the synthesis of uric acid from purine in the body and inhibit purine synthesis [2]. Despite the proven efficiency of these synthetic drugs in reducing and preventing gout diseases, the prolonged use has been causing serious side effects on gout patients, such as allergies, migraine, renal dysfunction and aseptic meningitis [13]. Due to such concern, efforts have been focused on searching effective and safe natural compounds from plants with higher therapeutics and fewer/no side effect to treat hyperuricemia.

*A. longiloba* (family *Araceae*) is locally known in Malaysia as ‘Keladi Candik’. The plant has been used by local traditional medicine practitioners for the treatment of wounds, furuncle and joint inflammation. The juice prepared from fruit and/or paste prepared from petiole are mostly used and externally applied on wounded skin to relive the painful inflammation and heal wounds. Latif et al. [14], reported that the juice prepared from the stem of *A. longiloba* could stops bleeding and promotes the healing process. Our research group previously reported that the petiole ethanolic extracts from *A. longiloba* showed a wound healing activity in rat model [15]. This current study designed to assess the hypothesis that *A. longiloba* extracts will have XO inhibitory activity, antioxidant properties and beneficial phytochemical compounds. To the best of our knowledge, xanthine oxidase inhibitory activity of *A. longiloba* for the treatment of gout has not been studied. Hence, the present study was conducted to evaluate the in vitro XOI activity, phytochemical and GC-MS analysis of *A. longiloba*.

## 2. Results and Discussion

### 2.1. Qualitative Phytochemical Screening

Phytochemical screening of *A. longiloba* plant extracts exhibited the presence of various group of phytochemical compounds. Most bioactives from *A. longiloba* petiole and fruits were efficiently extracted in ethanol, water and methanol > hexane > chloroform; while glycosides were extracted with all solvents, alkaloids from *A. longiloba* fruit were efficiently extracted with both ethanol and chloroform. Flavonoids from *A. longiloba* petiole and fruit were extracted with water, ethanol, methanol and hexane extracts, meanwhile it was absent in chloroform extract. Terpenoids and saponins were extracted from petiole and fruit with water, ethanol, methanol and hexane extracts and however, it was not detected in chloroform extracts. Tannins and steroids were only extracted with both ethanol and methanol solvents and absent in all other solvents extracted. Phenols from *A. longiloba* petiole and fruit were efficiently extracted with aqueous, ethanol, methanol extracts. In the current study, both petiole and fruit extracts contain various types of important phytochemical compounds among them are alkaloids, terpenoids and flavonoids, which have been found to be effective in the prevention and therapy of several ailments, including cancer, and also to have antioxidant, antimicrobial, antiviral, anti-gout, anti-inflammatory, and anti-arthritis [16,17]. Furthermore, flavonoids have been reported to possess the ability to act as active inhibitors of xanthine oxidase and also act as free radicals scavenging agent by donating hydrogen atoms to free radicals. *A. longiloba* contains high amount of flavonoids and could be used as a new alternative to synthetic gout-drugs with increased therapeutic activity and fewer side effects [18]. Phytochemicals are also used for various purposes, such as pharmaceuticals, agro-chemicals, food flavoring, fragrances, coloring agents, bio-pesticides and food additives. Hence, our result suggest the prospective utilization of fruit and petiole of *A. longiloba* to inhibit the activity of XO enzyme.

### 2.2. Quantification of Total Phenolic and Flavonoid Content

The total phenolic and flavonoids content in ethanolic petiole and fruit extracts of *A. longiloba* are shown in Table 1. The total phenolic content of the ethanolic petiole and fruit extracts, calculated from the calibration curve (R^2^ = 0.997), was 288.14 ± 4.919 and 512.84 ± 2.035 mg gallic acid equivalents/ g, respectively. The fruit extract showed significant highest amount of phenolic content than petiole extract. While, the total flavonoid content of petiole and fruit extracts calculated from calibration curve (R^2^ = 0.993) was 453.18 ± 2.525 and 438.18 ± 5.636 mg quercetin equivalents/g, respectively. The flavonoid content was slightly higher in petiole extract as compared to fruit extract. However, the differences in flavonoid content, were not found to be statistically significant. The phenolic and flavonoids are essential plant secondary metabolites and many research have reported on their effective anti-oxidative activities and pharmacological benefits that may be attributed to the antioxidant activity, anticancer, anti-bacteria, anti-inflammation [19,20,21]. Our findings suggested that both petiole and fruit are rich in phenolic and flavonoids contents, which could be the major contributor to XO inhibitions in the in vitro test.

### 2.3. DPPH and ABTS Free Radical Scavenging Properties of A. longiloba

DPPH radical scavenging assay has been used to assess the capacity of plant extracts to scavenge DPPH free radicals [22]. Antioxidant properties of ethanolic petiole and fruit extracts of *A. longiloba* using DDPH assay are shown in Table 1. The results showed that the IC_50_ values (the concentration with scavenging activity of 50%) were found to be 126.23 ± 0.52 and 137.66 ± 0.09 μg/mL for petiole and fruit extracts, respectively. Among the extract tested, fruit extract showed the highest anti-oxidant activity as compared to petiole extract. However, the differences in IC_50_, were not found to be statistically significant. This study revealed that both fruit and petiole extracts exhibited excellent DPPH free radical scavenging activity.

The ABTS free radical scavenging method has been widely used to assess the antioxidant activity of hydrogen-donating antioxidants. In the ABTS scavenging assay, both petiole and fruit extracts showed promising result. Fruit extracts exhibited the lowest IC_50_ value of 83.40 ± 0.057 μg/mL, followed by petiole extract with the IC_50_ value 88.30 ± 0.05 μg/mL (Table 1). While, a reference compound (ascorbic acid) exhibited the IC_50_ value of 26.6 μg/mL. Although the fruit extracts showed the best ABTS radical scavenging activity as a compared with petiole extract, but it is still lower than with those obtained using standard ascorbic acid. The high scavenging activity of fruit extracts for ABTS radical was probably due to higher phenolic hydroxyl and carboxyl groups, as confirmed by total phenolic and flavonoid content analysis.

It is widely reported that the antioxidant activity of plant extracts is related to the total phenolic content and total flavonoid content, as well as solvents system have different effects on phenolic, flavonoid contents and antioxidant activities [23]. Hyun et al. [23] report the strong correlation between total phenolic compounds and antioxidant activities in *Dendropanax morbifera* Léveille plant extracts. Similarly, Jing et al. [16] and Re et al. [24] report that flavonoids have antioxidant properties and also act as free radicals scavenging agents. Antioxidants plays a major role in preventing oxidation of molecules inside a cell and protecting healthy cell from damage caused by free radicals, unstable and highly reactive molecules that the human body produces as a byproduct of metabolism and other pressures. Therefore, protection against oxidative damage/cell death is the pivotal mechanism for preventing the occurrence of most diseases [3,4]. The current study, therefore, concluded that both petiole and fruit extracts from *A. longiloba* contains active constituents that are capable of scavenging DPPH and ABTS free radical in order to protect the cells from oxidation and cell damages.

### 2.4. Xanthine Oxidase Inhibitory Activity of A. longiloba Extracts

Xanthine oxidase inhibitory activity of *A. longiloba* extracts is shown in Table 2. Plant extracts exhibiting higher than 50% enzyme inhibition at concentration of (50 μg/mL) was only determined. A total of two plant extracts (petiole and fruit extracts) demonstrated substantial XO inhibitory activity (≥50% inhibition) at 50 μg/mL. Among the extracts tested, ethanolic petiole extract exhibited the highest XO inhibitory activity of 70.40 ± 0.05% at the concentration of 100 μg/mL with the IC_50_ value of 42.71 μg/mL, followed by ethanolic fruit extracts (61.44 ± 1.24%) activity at 100 μg/mL with the IC_50_ value of 51.3 μg/mL (Table 2). It had been reported that plant extracts exhibiting >50% xanthine oxidase inhibition at 50 μg/mL warranted further investigation [24]. In this study, the lowest IC_50_ value was 42.71 μg/mL, exhibited by petiole ethanolic extract which indicating that ethanolic petiole extract could inhibit 50% of xanthine oxidase activity at specified IC_50_ value. XOI activity of petiole extracts was slightly higher as compared with fruit extract. However, the difference was not found to be statistically significant (Table 2). Traditionally, both plant parts have been used by the local people in Kelantan, Malaysia, to treat wounds and joint inflammation. Interestingly, our results for *A. longiloba* are in accordance with the traditional uses. A study proved that a chemical compound, such as flavonoids, can interact with xanthine oxidase by competitively hindering the enzyme actions [24]. Similar reports show that active constituents found in plant extracts, such as alkaloids, flavonoids and phenolic compounds, which possess XO inhibiting properties [2]. In the present study, the reported XO inhibitory activity of *A. longiloba* extracts may attributed to due to the presence of those phytochemicals screened in the current experiment. Therefore, this study suggest that extracts from *A. longiloba* can be used as XOI agents.

In this study, comparison was made between the plant extracts in different extraction solvents to determine the potent plant-part extracts and extraction solvent. Interestingly, the most potent plant-part was petiole and fruit of *A. longiloba* as both extracts have demonstrated more than 50% inhibition at 50 μg/mL concentration. Therefore, both parts of the plant were chosen for further GC-MS analysis as they found to have higher XO inhibitory activity and lowest IC_50_ value. Investigation was also made to determine the efficacy of extraction solvents to extract the bioactive compounds that have high XOI activity. In this context, higher XOI activity was obtained using ethanolic solvent. While other solvents like water, methanol, hexane, ethyl acetate and chloroform gave lower XOI activity. Therefore, ethanol was found to be optimal extraction solvent for *A. longiloba*. Jayawardena et al. [25], reported that extraction solvent is the most important factor that affect the extraction efficacy of bioactive compounds from plant. Dailey et al. [26], investigated the effect of extraction solvents on recovery of bioactive compound and antioxidant property. Their result shows that solvents has direct effect on the recovery, yield and therapeutics properties of bioactive compounds. Therefore, choosing proper solvent system for the extraction of bioactive compound is crucial. In this study, comparison was also made between ethanolic petiole and fruit extracts with allopurinol (positive control) in order to determine the potency of extracts, as compared with synthetic drugs. This results shows that both petiole and fruit extracts have shown promising XOI activity, even though their activity is slightly lower than allopurinol.

The anti-gout property and XOI activity by plant extracts has been demonstrated by researchers [27,28]. Alsultanee et al. [28], investigated XOI activity of methanolic extract of *Cucurbitaceae* and their results showed that extract were effective against XO. Similarly, Yumita et al. [27] studied XOI activity of *Alpinia galanga* Linn and *Woodfordia floribunda* Salisb and they ascertained that *A. galanga* Linn extracts had the highest XOI activity. Alsultanee et al. 2014 [28], investigated XOI potential of extract of *Carica papaya*. Their result showed promising XOI activity. Several researchers have established the therapeutic properties and constituents of medicinal plants and various plant species has been reported to have antioxidant and free radical scavenging activity. Among different kinds of plant constituents (phytochemicals), flavonoid compounds have been reported to have the greatest therapeutic properties to treat most of ailments. The phenolic and flavonoid compounds are a main class of plant secondary metabolites identified by the presence of aromatic ring at least one hydroxyl group. Phenolic compounds have a good electron-donating ability because of their hydroxyl groups that can directly contribute to antioxidant activity. These plant constitutes or substances can be stored in one or more of its organ including fruit, leaves, petiole, root, tuber, bark and flowers. Different organ (tissues) of plant may contain different active ingredients within the same plant [27,28]. Extract of *A. longiloba* could be favored for its XO inhibitory effect than synthetic drugs as plant product is regarded as safe herbal medicines and possess less or no side effect. Hence, our result suggest the prospective utilization of fruit and petiole of *A. longiloba* to inhibit the activity of XO enzyme.

### 2.5. Gas Chromatography-Mass Spectrometry (GC-MS) Analysis

**Figure 1:**
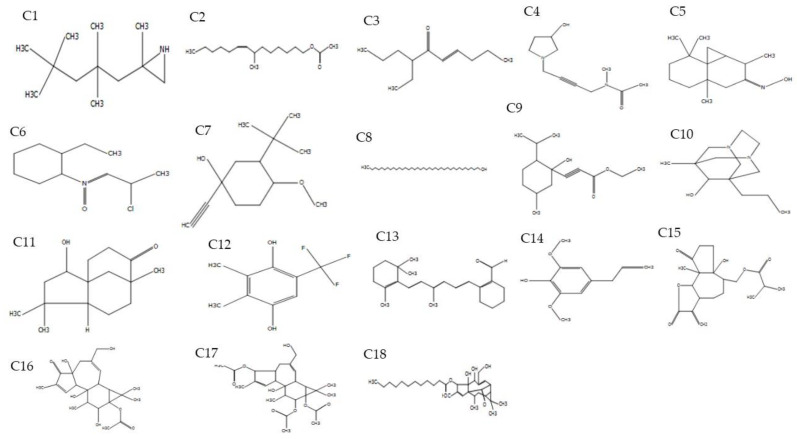
Chemical structures of compounds identified in ethanolic extract of *A. longiloba* petiole. (C1-C18). * C-compound.

**Figure 2:**
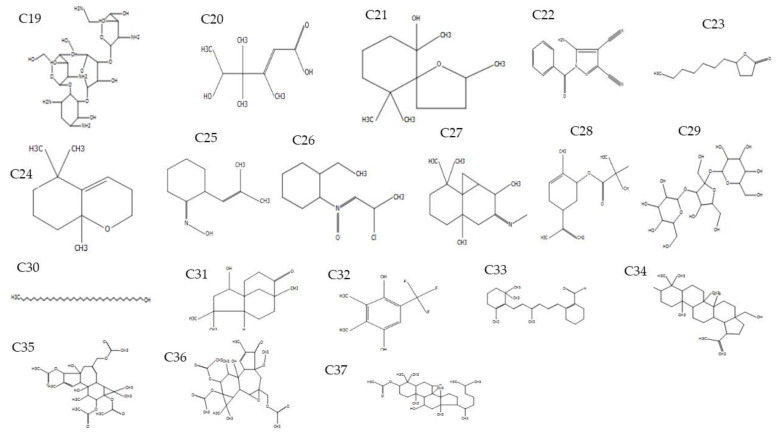
Chemical structures of compounds identified in ethanolic extract of *A. longiloba* fruit. (C19-C37). * C-compound.

GC-MS analysis of petiole and fruit extracts of *A. longiloba* revealed the presence of various groups of bioactive compounds. The bioactive compounds with their retention time (RT), molecular formula, molecular weight (MW), ion mass (*m*/*z*) and biological activity are exhibited in Table 3. The molecular structure of the bioactive phytoconstituents are showed in Figure 1 and Figure 2 (see Supplemental Materials).

Aziridine,2-methyl-2-(2,2,4,4-tetramethylpentyl), 7-Methyl-*Z*-tetradecen-1-olacetate, 7-Ethyl-4-decen-6-one, Acetamide,*N*-methyl-*N*-[4-(3-hydroxypyrrolidinyl)-2-buty, Cyclopropa[d]naphthalen-3-one,octahydro-2,4a,8,8-tetramethyl-,oxime, octahydro-2,4a,8,8, 2-Ethylcyclohexylamine,*N*-(2-chloropropylidene)-,Noxide, 1-Ethynyl-3,trans(1,1-dimethylethyl)-4,cismethoxycyclohexan-1-ol, 1-Heptatriacotanol, Propiolicacid,3-(1-hydroxy-2-isopropyl-5-methylcyclohexyl),ethylester, 1-Methyl-8-propyl-3,6-diazahomoad, 2-Hydroxy-4,4,8-trimethyltricyclo, Phen-1,4-diol, 2,3-dimethyl-5-trifluoromethyl-, 2-[4-methyl-6-(2,6,6-trimethylcyclohex-1-enyl), Phenol, 2,6-dimethoxy-4-(2-propenyl, Propanoicacid,2-methyl-,(dodecahydro-6a-hydroxy-9amethyl-3-methylene, 5H-Cyclopropa[3,4]benz[1,2-e]azulen, 1*H*-Cyclopropa[3,4]benz[1,2-e]azulene, Dodecanoic acid were present in the ethanolic extracts of *A. longiloba* petiole. In the present study, some of the identified compounds has been reported to have various biological activities. For instance, 1-Ethynyl-3,trans(1,1-dimethylethyl)-4, 7-Methyl-Z-tetradecen and 1-Heptatriacotanol possesses anti-inflammatory, anti-cancer and anti-oxidant activity [29,30].

**Figure 3:**
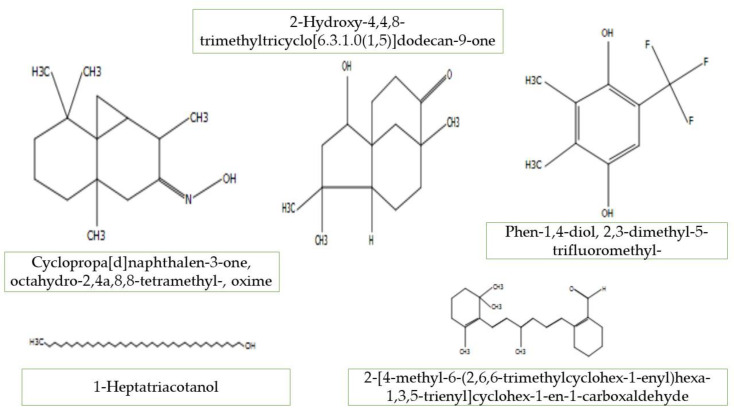
The chemical structures of GC-MS detected compounds in both fruit and petiole.

Paromomycin, 2-Hexenoic, 5-hydroxy-3,4,4-trimethy, 2,6,10,10-Tetramethyl-1-oxaspiro, 5-Amino-1-benzoyl-1*H*pyrazole-3,4-dicarbonitrile, 2(3*H*)-Furanone, 5-heptyldihydro, 5,5,8a-Trimethyl-3,5,6,7,8,8a-hexahydro-2*H*chromene, 2-(2-Methyl-propenyl)-cyclohexanoneoxime, 2-Ethylcyclohexylamine,*N*-(2-chloropropylidene)-,Noxide, Cyclopropa[d]naphthalen-3-one, octahydro-2,4a,8,8-tetramethyl-,oxime, Limonen-6-ol,pivalate, α-DGlucopyranoside,*O*-α-d-glucopyranosyl-(1.fwdarw.3)-β-Dfructofuranosyl, 1-Heptatriacotano, 2-Hydroxy-4,4,8-trimethyltricyclo[6.3.1.0(1,5)]dodecan-9-one, Phen-1,4-diol, 2,3-dimethyl-5-trifluoromethyl-, 2-[4-methyl-6-(2,6,6-trimethylcyclohex-1-enyl)carboxaldehyde, Betulin, 1*H*Cyclopropa[3,4], 3-[(acetyloxy)methyl]-1b,4,5,7a,8,9-hexahydro-1,1,6,8-tetra, 4HCyclopropa[5′,6′]benz were present in the ethanolic extracts of *A. longiloba* fruit. The identified compounds from fruit extract have been reported to have therapeutics properties by several researchers. For example, Paromomycin, Limonen-6-ol, pivalate, α-DGlucopyranoside,*O*-α-d-glucopyranosyl-(1.fwdarw.3)-β-Dfructofuranosyl, Phen1,4-diol,2,3-dimethyl-5-trifluoromethyl, and Betulin possesses anti-biotic, anti-inflammatory, anti-carcinogenic, ant-microbial activity [30,31,32]. In this study, some phytochemical compounds were detected in both, ethanol fruit and petiole extracts such as Cyclopropa[d]naphthalen-3-one,octahydro-2,4a,8,8-tetramethyl-,oxime, 2-Hydroxy-4,4,8-trimethyltricyclo[6.3.1.0(1,5)]dodecan-9-one, Phen-1,4-diol,2,3-dimethyl-5trifluoromethyl-, 1-Heptatriacotanol, and 2-[4-methyl-6-(2,6,6-trimethylcyclohex-1-enyl)hexa-1,3,5-trienyl]cyclohex-1-en-1-carboxaldehyde, this indicates the richness levels in both plant tissues (Figure 3). The presence of various bioactive compounds detected after GC-MS analysis using the ethanolic extracts of *A. longiloba* justifies the use of this plant for various elements by traditional medicine practitioners. Yet, isolation of individual phytoactive compounds and subjecting to scientific study will be useful.

## 3. Materials and Methods

### 3.1. Chemicals and Equipment

All the chemical used in these experiment were analytical grade. Allopurinol, xanthine (substrate solution), xanthine oxidase (butter milk), sodium phosphate monobasic dehydrate and disodium hydrogen phosphate, hydrochloric acid (HCl), sulfuric acid, sodium hydroxide, acetic acid, potassium persulfate, Wager reagent, Mayer reagent, sodium carbonate, aluminum chloride, ferric chloride, gallic acid, quercetin, Folin-Ciocalteu reagent, DPPH, ABTS, ascorbic acid, were procured from Sigma-Aldrich. Solvents such as ethanol, methanol, chloroform, ethyl acetate, hexane, DMSO and distilled water were obtained from Merck (Darmstadt). Heavy duty electrical blender (Milux), Aldrich-Soxhlet (Germany) extractor and cellulose thimbles (41 mm × 123 mm) were used for the extraction of compounds. Rotary evaporator (R-200) were used to evaporate solvents from the extracts. UV-Spectrophotometer (Thermo scientific Genesys 20) and spectrophotometer cuvettes were used.

### 3.2. Plant Material Collection

*A. longiloba* plant were collected from Kota Bahru, Kelantan, Malaysia (6.1211° N, 102.3178° E). The authenticity of the plant was verified by Zulhazman Hamzah at Faculty of Earth science, University Malaysia Kelantan, Malaysia. The plant tissues such as fruit and petiole were carefully separated from the plant and washed under running water for 30–45 min to remove the dusts present in the plant material. The plant part were further given cut in to small pieces to enhance the drying process and then dried under the oven for 72 h at 40 °C.

### 3.3. Preparation of Extracts

The dried plant tissues such as fruit and petiole were grounded into fine powder using electrical blender (Milux MFP-9625 heavy duty blender). The powdered material was extracted following the method by Edeoga et al. [36]. The solid to solvent ratio of 1:10 (*w*/*v*), briefly thirty gram of dried powdered sample was added into 300 mL solvent in 500 mL round bottom flasks for soxhlet extraction (6 cycles). In this study, five extraction solvents were employed based on solvents polarity, namely 95% ethanol, methanol, hexane, chloroform and distilled water. The solvent was evaporated under vacuum at 35–40 °C through a rotary evaporator to concentrate the product. The solid residues were collected and stored in the freezer at 4 °C for further use.

### 3.4. Qualitative Phytochemical Screening

The ethanol, methanol, hexane, chloroform and distilled water extracts of *A. longiloba* subjected to examination for the detection of phytochemical compounds following the procedure of Njoku et al. [37] and Ayoola et al. [38]. The qualitative analysis of various phytochemicals was carried out by using Mayer’s and Wagner’s reagents (Alkaloids). Other tests carried out, include the foam test (saponins) and test for glycosides. Salkowski test carried out to detect steroids and triterpenoids. Ammonia solution (flavonoids) test and ferric chloride used to detect phenols and tannins compounds [38].

### 3.5. Quantification of Total Phenolic and Total Flavonoid Content

The total phenolics content of ethanolic petiole and fruit extracts were determined using Folin-Ciocalteu reagent following the procedure by Hyun et al. [23] with minor modification. Briefly, 100 μL of different concentrations of test sample was mixed with 1 mL of diluted FC reagent (1:10). After 10 min, 1 mL of 7.5% (*w*/*v*) sodium carbonate solution was added to the mixture and incubated in the dark for 90 min. The absorbance was recorded at 725 nm. The phenolic content was calculated as from calibration curve and expressed as mg of gallic acid equivalents per gram of dry weight (mg GA/g extract).

The total flavonoid content was determined by the aluminum chloride colorimetric method following the procedure by Jing with a minor modification [16]. Briefly, 50 μL of 5% (*w*/*v*) sodium nitrate solution was added to 0.5 mL of various concentrations of extract and then it was allowed to react for 5 min. Then, 50 μL of 10% (*w*/*v*) aluminum chloride solution was added. After 5 min, 250 μL of 4% (*w*/*v*) NaOH were added into the mixture. The absorbance was measured at 518 nm immediately. Quercetin was used to make standard calibration curve. The concentration of total flavonoid was expressed as mg of quercetin equivalents per g of dry extract (mg QCE/g extract).

### 3.6. Determination of Antioxidant Activity

#### 3.6.1. DPPH (1,1-Diphenyl-2-picryl-hydrazyl) Assay

The ability of plant extract to scavenge the DPPH free radicals was determined following the method of Kabir et al. [5] with minor modifications. Briefly, 2 mL of test sample (12.5, 25, 50, 100, 200 and 400 μg/mL) were mixed with 2 mL 0.004% *w*/*v* DPPH solution dissolved in MeOH and then incubated in the dark for 30 min. The absorbance was recorded at 517 nm. Ascorbic acid was used as a positive control. The capacity of the extracts to scavenge free radicals was calculated using the equation below Equation (1).

DPPH scavenging effect (%) = (A_1_ − A_0_/A_1_) × 100 (1)

where A_1_ was the absorbance of control (DPPH solution only); A_0_ was the absorbance of test extract at various concentrations with DPPH.

#### 3.6.2. ABTS (2,2′-azinobis-(3-ethylbenzothiazoline-6-sulfonate) Radical Scavenging Assay

The antioxidant activity of test sample to scavenge the ABTS radicals was determined following the method of Re et al. [24] with some modifications. ABTS solution was prepared by mixing equal volumes of 7 mM ABTS with 2.45 mM potassium persulfate. Then mixture allowed to stand in the dark at room temperature for 16 h. This solution was suitably diluted with methanol to yield an absorbance of 0.701 ± 0.03 at 734 nm and then used for anti-oxidant assay. Briefly, 1 mL of different concentrations of extract (12.5, 25, 50, 100, 200 and 400 μg/mL) were added to 2 mL of to the above activated pre-generated ABTS solution and the vortexed for 1 min. After 10 min incubation in the dark, then the absorbance was measure at 734 nm, using methanol as a blank. The result was compared with control (only ABTS solution) having absorbance 0.701 ± 0.021. ABTS radicals scavenging activity was calculated using the formula Equation (2)

ABTS scavenging effect (%) = (A_1_ − A_0_/A_1_) × 100 (2)

where A_1_ was the absorbance of control (ABTS solution only); A_0_ was the absorbance of test extract at various concentrations with ABTS.

### 3.7. Xanthine Oxidase Inhibitory Activity In Vitro Assay

XOI activity was conducted following the procedure of Azmi et al. [39] with some modification. XOI activity was determined based on measuring uric acid formation spectrometrically at 295 nm using UV spectrophotometer. The assay mixture consisted of 300 µL of phosphate buffer (pH 7.5), 100 µL of plant extracts (concentration range: 50, 100, 150 and 200 µg/mL) prepared in 1% DMSO, 100 μL XO enzyme solution (0.2 units/mL in phosphate buffer, pH 7.5 at 25 °C), and 100 μL of distilled water, all the solution were prepared freshly. After pre-incubation at 37 °C for 15 min, the reaction was initiated by the adding 200 μL of xanthine substrate solution (0.15 mM) into the mixture. The mixture was incubated at 37 °C for 30 min. Finally, the reaction was then stopped by addition of 200 μL of 0.5M hydrochloric acid. Absorbance was taken at 295 nm. Allopurinol was used as positive control. XOI activity of assayed samples expressed as percentage inhibition of XO. A blank was prepared in the same way, however the XO were not added in blank solution. The inhibition percentage calculated using the formula Equation (3)

% XO inhibition = (1 − β/α) × 100(3)

where, α is XO activity without extract and *β* is XO activity with extract and the results were expressed in µg/mL. Based on the value inhibition percentage at various concentrations, the IC_50_ values were determined.

### 3.8. Gas Chromatography–Mass Spectrometry (GC–MS) Analysis

The GC-MS analysis was performed using Agilent Technologies 7693 and 5977 A MSD following the procedure adopted by Sermakkani et al. [40]. Ethanolic plant extracts were filtered through purple nylon syringe filter (0.45 μm) and then two microliter of plant extract was injected in the split mode (10:1). Helium gas was used as a carrier, at the rate of 1 mL/min. The injector temperature was 250 °C. Then analytes were separated on a fused silica capillary column (30 m × 0.25 mm × 1 μm). The oven program was set as follows: initial temperature of 110 °C held for 2 min, and then ramped to 200 °C at a rate of 10 °C/min without holding; 280 °C was maintained for 9 min with program rate of 5 °C /min. For mass spectra determination, ionization energy of 70 eV, while mass scanning range was 10–400 *m/z*. Identification and interpretation of GC-MS mass spectrum was conducted using NIST library mass spectra. Furthermore, the retention index (RI), name, molecular structure and weight of the components of the test extracts were ascertained with those obtained in the literature.

### 3.9. Statistical Analysis

The IC_50_, the concentration of a plant extracts that is required for 50% enzyme activity and free radicals inhibition, were calculated through linear regression analysis. Student’s *t*-test were employed and significant difference were stablished at *p* < 0.05. SPSS version 13.0 software and Minitab version 15.0 statistical software were used to carry out regression analysis.

## 4. Conclusions

In this work, a comprehensive study on in vitro xanthine oxidase inhibitory, antioxidant activities, phytochemical screening and GC-MS profile of *A. longiloba* was performed for the first time. The results of the current study support the original hypothesis. The petiole and fruit extracts possess various groups of phytochemicals with high total phenolic and flavonoids content, as well as potential antioxidant property. The extracts were able to inhibit the activity of XO enzyme in a concentration dependent manner. Moreover, the GC-MS analysis of both extracts revealed the presences of various phytoconstituents that have been known to possess therapeutics properties. From these results, it could be concluded that *A. longiloba* possess various bioactive compounds, strong antioxidant and XOI properties. Yet, further in vivo studies are necessary to validate the reported in vitro XO inhibitory and antioxidant activities in animal model.

## Figures and Tables

**Table 1:** Total phenolic and flavonoids content and Antioxidant activity of ethanolic extracts of *A. longiloba*.

Total Amount/Scavenging Activity	Extracts	
	Petiole	Fruit
Total Phenolic Content mg GAE/g	288.14 ± 4.91 ^b^	512.84 ± 2.03 ^a^
Total Flavonoids Content mg QCE/g	453.18 ± 2.52 ^a^	438.18 ± 5.63 ^a^
DPPH, IC_50_ (μg/mL)	126.23 ± 0.52 ^a^	137.66 ± 0.09 ^a^
ABTS, IC_50_ (μg/mL)	88.30 ± 0.05 ^a^	83.40 ± 0.057 ^a^

Values are presented as mean ± standard error (*n* = 3). The means with different lowercase letters (a, b) in the same column are significantly different at *p <* 0.05 (ANOVA, followed by Duncan’s multiple comparison test).

**Table 2:** Xanthine oxidase inhibitory activity of ethanolic petiole and fruit extract of *A. longiloba.*

Concentration (μg/mL)	Percent of Inhibition (%) of Plant Extracts	
	Petiole	Fruit
50	54.63 ± 1.44	50.33 ± 0.56
100	70.40 ± 0.05	61.44 ± 1.24
150	73.76 ± 0.95	65.53 ± 0.86
200	77.03 ± 0.95	70.40 ± 0.10
250	85.76 ± 1.08	75.96 ± 2.41
IC_50_ (μg/mL)	42.71 *	51.32 *

Values are presented as mean ± standard error (*n* = 3). * No significant difference was shown between extracts after testing in the independent *t*-test.

**Table 3:** List of compounds from ethanolic petiole and fruit extracts observed in GC-MS with their retention time and biological activity.

Compound	Name of Compound	Plant Part	RT (min)	Peak Area %	Ion Mass (m/z)	M.F	M.W	Biological Activity
1	Aziridine,2-methyl-2-(2,2,4,4-tetramethylpentyl)-	Petiole	3.52	3.25	124.0	C_12_H_25_N	183	Not reported
2	7-Methyl-*Z*-tetradecen-1-ol acetate	Petiole	4.06	1.65	126.02	C_17_H_32_O_2_	268	Anti-cancer, anti-inflammatory, [29]
3	7-Ethyl-4-decen-6-one	Petiole	5.55	100	110.0	C_12_H_22_O	182	Not reported
4	Acetamide,*N*-methyl-*N*-[4-(3-hydroxypyrrolidinyl)-2-butynyl]-	Petiole	6.15	17.85	124.01	C_11_H_18_N_2_O_2_	210	Anti-fungal activity [33]
5	Cyclopropa[d]naphthalen-3-one,octahydro-2,4a,8,8-tetramethyl-,oxime	Petiole	6.67	33.18	124.02	C_15_H_25_NO	235	Not reported
6	2-Ethylcyclohexylamine,*N*-(2-chloropropylidene)-,*N*-oxide	Petiole	7.16	17.28	154.08	C_11_H_20_ClNO	217	Not reported
7	1-Ethynyl-3,trans(1,1-dimethylethyl)-4,cis-methoxycyclohexan-1-ol	Petiole	7.50	5.91	97.0	C_13_H_22_O_2_	210	Anti-Candida, anti-inflammatory [29]
8	1-Heptatriacotanol	Petiole	7.76	8.14	123.02	C_37_H_76_O	536	Antioxidant, anticancer and, anti inflammatory [30]
9	Propiolic acid, 3-(1-hydroxy-2-isopropyl-5-methylcyclohexyl)-, ethyl ester	Petiole	9.12	4.42	191.11	C_15_H_24_O_3_	252	Antiangiogenic activity [30]
10	1-Methyl-8-propyl-3,6-diazahomoadamantan-9-ol	Petiole	9.30	5.19	137.04	C_13_H_24_N_2_O	224	Not reported
11	2-Hydroxy-4,4,8-trimethyltricyclo[6.3.1.0(1,5)]dodecan-9-one	Petiole	9.79	8.85	180.10	C_15_H_24_O_2_	236	Not reported
12	Phen-1,4-diol, 2,3-dimethyl-5-trifluoromethyl-	Petiole	10.12	5.22	149.0	C_9_H_9_F_3_O_2_	206	Antioxidant, antithrombotic and anti-tuberculosis [29]
13	2-[4-methyl-6-(2,6,6-trimethylcyclohex-1-enyl)hexa-1,3,5-trienyl]cyclohex-1-en-1-carboxaldehyde	Petiole	11.15	4.09	73.0	C_23_H_32_O	324	Not reported
14	Phenol, 2,6-dimethoxy-4-(2-propenyl)-	Petiole	11.36	4.45	194.10	C_11_H_14_O_3_	194	Anti-fungal and Anti-helminthic
15	Propanoic acid, 2-methyl-, (dodecahydro-6a-hydroxy-9a-methyl-3-methylene-2,9-dioxoazuleno[4,5-b]furan-6-yl)methyl ester,	Petiole	12.57	6.01	135.04	C_19_H_26_O_6_	350	Anti-biotic
16	5H-Cyclopropa[3,4]benz[1,2-e]azulen-5-one,	Petiole	17.63	1.69	81.04	C_28_H_36_O_11_	548	Not reported [29]
17	1H-Cyclopropa[3,4]benz[1,2-e]azulene-5,7b,9,9a-tetrol, 1a,1b,4,4a,5,7a,8,9-octahydro-3-(hydroxymethyl)-1,1,6,8-tetramethyl-,	Petiole	18.36	4.51	91.08	C_26_H_36_O_8_	476	Not reported
18	Dodecanoic acid, 1a,2,5,5a,6,9,10,10a-octahydro-5,5a-dihydroxy-4-(hydroxymethyl)-1,1,7,9-tetramethyl-11-oxo-1H-2,8a-methanocyclopenta	Petiole	19.65	1.95	105.06	C_32_H_50_O_6_	530	Flavor [34]
19	Paromomycin	Fruit	3.13	3.31	112.02	C_23_H_45_N_5_O_14_	615	Antibiotic [32]
20	2-Hexenoic acid, 5-hydroxy-3,4,4-trimethyl-, (*E*)-	Fruit	3.52	7.91	128.0	C_9_H_16_O_3_	172	Not reported
21	2,6,10,10-Tetramethyl-1-oxaspiro[4.5] decan-6-ol	Fruit	4.21	3.18	85.0	C_13_H_24_O_2_	212	Not reported
22	5-Amino-1-benzoyl-1H-pyrazole-3,4-dicarbonitrile	Fruit	4.97	3.18	122.0	C_12_H_7_N_5_O	237	Not reported
23	2(3H)-Furanone, 5-heptyldihydro	Fruit	5.16	1.8	85.0	C_11_H_20_O_2_	184	Not reported
24	5,5,8a-Trimethyl-3,5,6,7,8,8a-hexahydro-2*H*-chromene	Fruit	6.16	5.18	124.03	C_12_H_20_O	180	Not reported
25	2-(2-Methyl-propenyl)-cyclohexanone oxime	Fruit	6.67	2.85	124.02	C_10_H_17_NO	167	Not reported
26	2-Ethylcyclohexylamine, *N*-(2-chloropropylidene)-, *N*-oxide	Fruit	7.00	1.21	154.08	Not found		Not reported
27	Cyclopropa[d]naphthalen-3-one, octahydro-2,4a,8,8-tetramethyl-, oxime	Fruit	7.75	4.34	142.01	C_15_H_25_NO	235	Not reported
28	Limonen-6-ol, pivalate	Fruit	8.87	11.42	133.02	C_15_H_24_O_2_	236	Antioxidant and anti-inflammatory [30]
29	α-DGlucopyranoside,*O*-α-d-glucopyranosyl-(1.fwdarw.3)-β-d-fructofuranosyl	Fruit	9.15	19.39	137.06	C_18_H_32_O_16_	504	Anticarcinogenic, and anti-microbial [30]
30	1-Heptatriacotanol	Fruit	9.32	1.68	163.08	C_37_H_76_O	536	Antioxidant, anticancer and inflammatory [30]
31	2-Hydroxy-4,4,8-trimethyltricyclo[6.3.1.0(1,5)]dodecan-9-one	Fruit	9.80	3.28	180.10	C_15_H_24_O_2_	236	Not reported
32	Phen-1,4-diol, 2,3-dimethyl-5-trifluoromethyl-	Fruit	10.12	1.95	149.0	C_9_H_9_F_3_O_2_	206	Antimicrobial activity [35]
33	2-[4-methyl-6-(2,6,6-trimethylcyclohex-1-enyl)hexa-1,3,5-trienyl]cyclohex-1-en-1-carboxaldehyde	Fruit	12.66	3.99	123.10	C_23_H_32_O	324	Antimicrobials and antiviral [35]
34	Betulin	Fruit	14.77	1.29	267.10	C_30_H_50_O_2_	442	Anti-inflammatory, and cytotoxicity [31]
35	1*H*-Cyclopropa[3,4]benz[1,2-e]azulene-4a,5,7b,9,9a(1aH)-pentol, 3-[(acetyloxy)methyl]-1b,4,5,7a,8,9-hexahydro-	Fruit	15.63	1.55	69.10	C_28_H_38_O_10_	534	Not reported
36	4*H*-Cyclopropa[5′,6′]benz[1′,2′:7,8]azuleno[5,6]oxiren-4-one, 8,8a-bis(acetyloxy)-2a-[(acetyloxy)methyl]-1,1a,1b,1c,2a,3,3a,6a	Fruit	19.04	1.23	69.10	C_27_H_36_O_10_	520	Not reported
37	7,8-Epoxylanostan-11-ol, 3-acetoxy-	Fruit	20.37	3.66	139.10	C_32_H_54_O_4_	502	Not reported

* RT—retention time; M.F—molecular formula; M.W—molecular weight.

## Supplementary Materials

Supplementary Materials depicts tissue specific compounds (compound 1 in petiole and compound 19 in fruit).
