# Supplementary figures and images for: Xanthine Oxidase Inhibitory Activity, Chemical Composition, Antioxidant Properties and GC-MS Analysis of Keladi Candik (Alocasia longiloba Miq)
Source: Molecules. 2020 Jun 8;25(11):2658. doi: 10.3390/molecules25112658 (PMC7321287; doi:10.3390/molecules25112658)

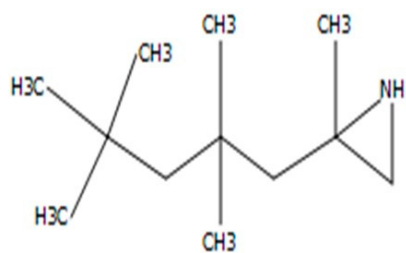

Aziridine, 2-methyl-2-(2,2,4,4-tetramethylpentyl)-

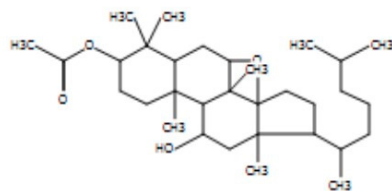

7,8-Epoxy lanostan-11-ol, 3-acetoxy-

Supplement: Supplementary file 1 [file molecules-25-02658-s001.pdf]
